# Retroperitoneal neuroglial heterotopia in infancy: case report and review of literature

**DOI:** 10.1093/jscr/rjaf893

**Published:** 2025-11-14

**Authors:** Hemonta Kumar Dutta, Rishiraj Baruah, Pallav Chowlu, Preetam Kr Das, Diganta Saikia

**Affiliations:** Department of Pediatric Surgery, Assam Medical College, PO Borbari, District-Dibrugarh 786002, India; Madonna Diagnostic & Research Centre, PO Bordoloi Avenue, District-Dibrugarh 786002, India; Department of Pediatric Surgery, Assam Medical College, PO Borbari, District-Dibrugarh 786002, India; Department of Pediatric Surgery, Assam Medical College, PO Borbari, District-Dibrugarh 786002, India; Department of Anaesthesiology, Assam Medical College, PO Borbari, District-Dibrugarh 786002, India

**Keywords:** neuroglial heterotopia, retroperitoneal neuroglial heterotopias, retroperitoneal tumour

## Abstract

Neuroglial heterotopia (NH) is a rare entity which is composed of displaced masses of mature central neuroepithelial tissue manifesting in locations unconnected to the brain and spinal cord. NH is reported in the head and neck region, most commonly in the nose and nasopharynx, but some other rare sites, such as lung, extremities, and skin are also reported. NH presenting in the retroperitoneum and during infancy is extremely rare. We present a case of an infant who presented with lump abdomen and respiratory tract infection. Imaging revealed a large retroperitoneal tumour, which was safely excised. Histopathology revealed features of NH which was further confirmed by immunohistochemistry.

## Introduction

Neuroglial heterotopia (NH) is a rare, benign, congenital anomaly in which mature neuroepithelial tissue is found in an ectopic location outside the skull or spinal canal [1]. NH is usually found in the head and neck region, most commonly in the nose and nasopharynx and is also reported in other sites such as lung, extremities, and skin. NH is extremely rare in retroperitoneal location and only few cases are reported in English literature [2]. In this report, we present an infant who presented with an abdominal lump and symptoms of upper respiratory tract infection (RTI). After surgical excision, histopathology and immunohistochemical studies, it was reported as retroperitoneal NH.

## Case report

**Figure 1:**
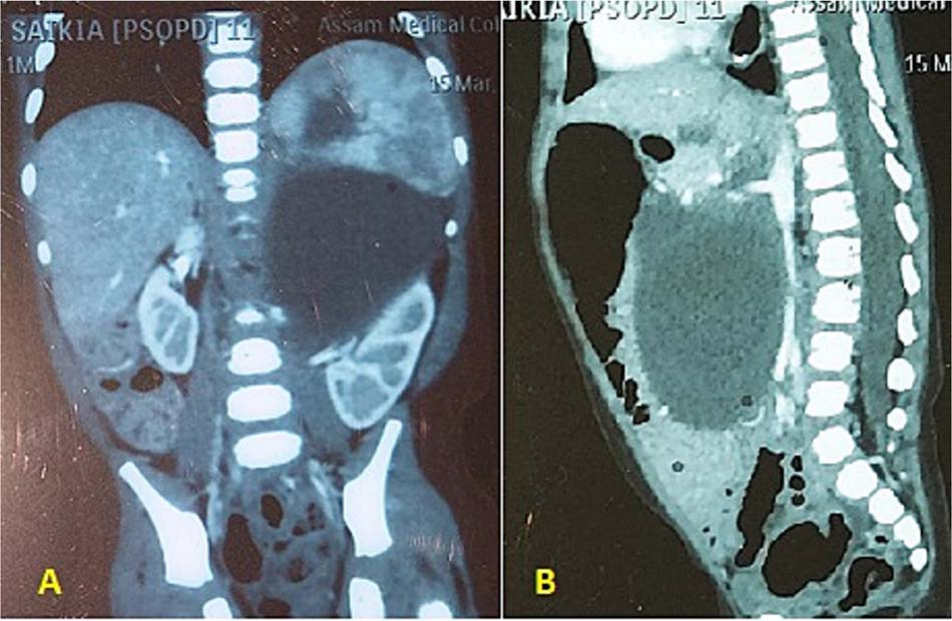
(A, B) CECT abdomen shows multicystic/solid mass pushing the abdominal organs.

**Figure 2:**
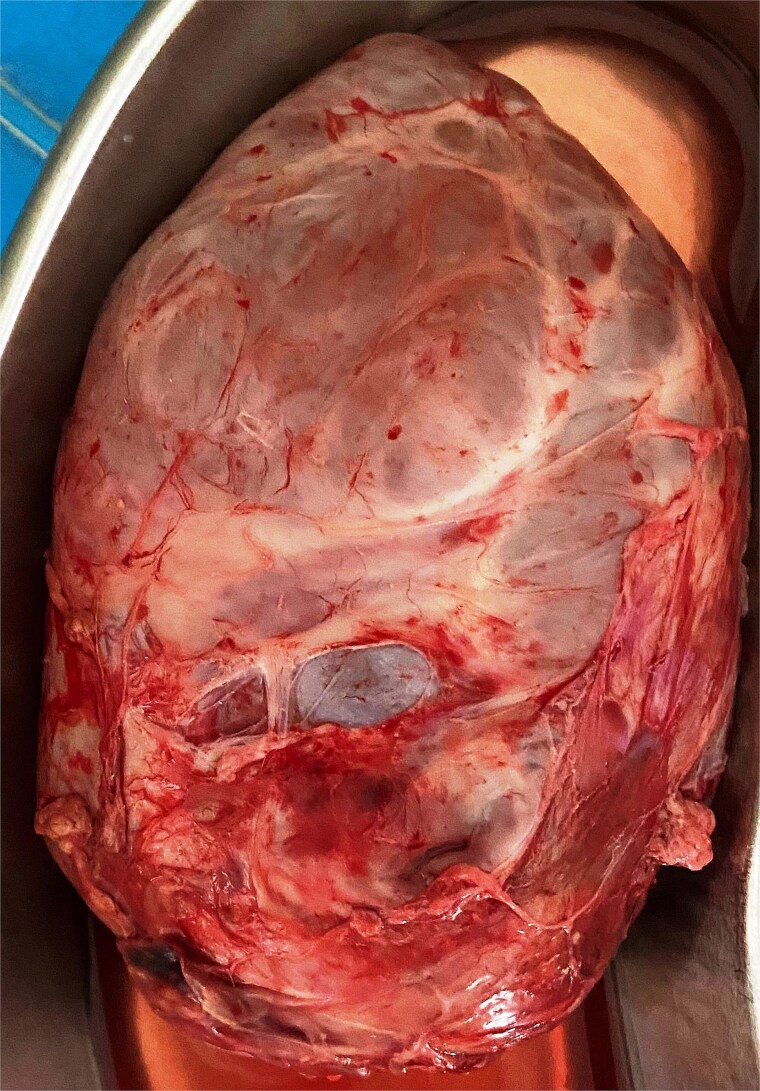
The excised tumour with variable size cysts and solid component.

An 11 and half month old female child born out of a non-consanguineous marriage presented with lump abdomen and recurrent upper RTI of 3 months duration. The child was breast fed till 7 months of age and then supplemented with rice, dal, etc. The baby had normal weight gain till about 7 months (9.5 kg), but recorded 8.8 kg at the time of presentation. No urinary or bowel symptoms were present. The parents noted a lump in the abdomen at 7 months of age, which gradually kept increasing. An ultrasonography revealed a large multiloculated cystic lesion of 14 × 9 cm dimension in the retroperitoneum. Her haemoglobin level was 10.1 gm%. Other blood parameters were within normal range. Chest X-ray was normal. A contrast CT scan showed a multiloculated cystic lesion with thick septations displacing abdominal organs in the left retroperitoneum (Fig. 1A and B). Serum β-HCG level was within normal range and α-fetoprotein level was marginally raised. The child was admitted and operated through a left transverse upper abdominal incision. The left colon was mobilized and retracted medially; the pancreas, stomach, and spleen were retracted. The vessels supplying the tumour from major vessels were divided between ligatures. The tumour was mostly cystic with solid elements located posterior-superiorly. The tumour could be mobilized from the left kidney and adrenal, the spleen, pancreatic tail, and pelvic organs and was excised safely. There was no connection with the spine. The abdomen was closed without a drain. The mass weighed 550 g and had cysts of variable sizes with mucoid content (Fig. 2). Solid elements had soft tissues without any bone or haemorrhagic area. Hundred millilitre of packed cell was infused post-surgery. The patient was allowed feed 12 h post-op, had an uneventful recovery and was discharged on post-op day 4. She has been on follow-up since then and has normal activity and weight gain.

**Figure 3:**
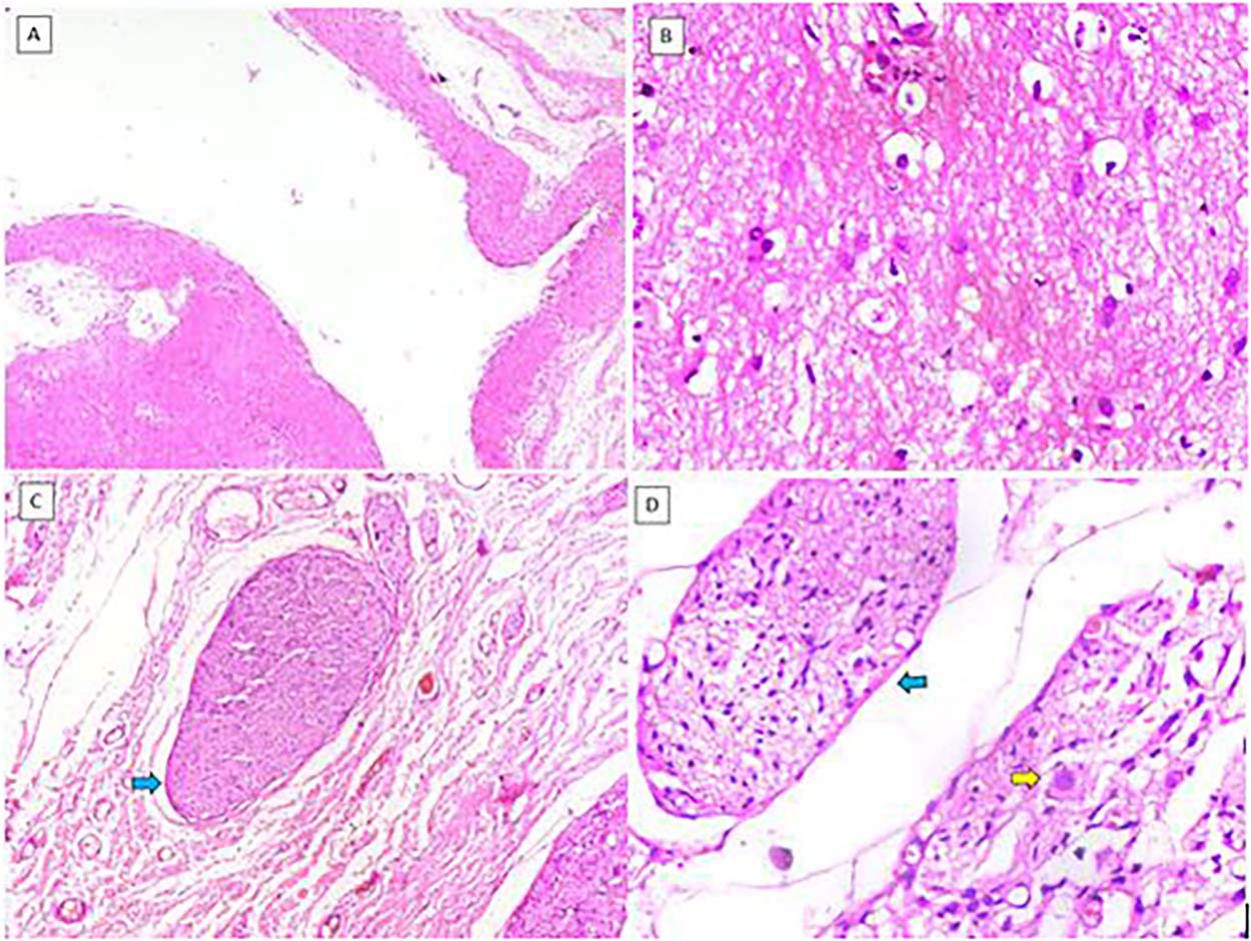
(A) Cystic structure lined by neuroglial tissue (H&E, 40×). (B) Glial tissue (H&E, 400×). (C) Nerve trunks (blue arrow) (H&E, 100×). (D) Nerve trunks (blue arrow) and ganglion cells (yellow arrow) (H&E, 400×).

**Figure 4:**
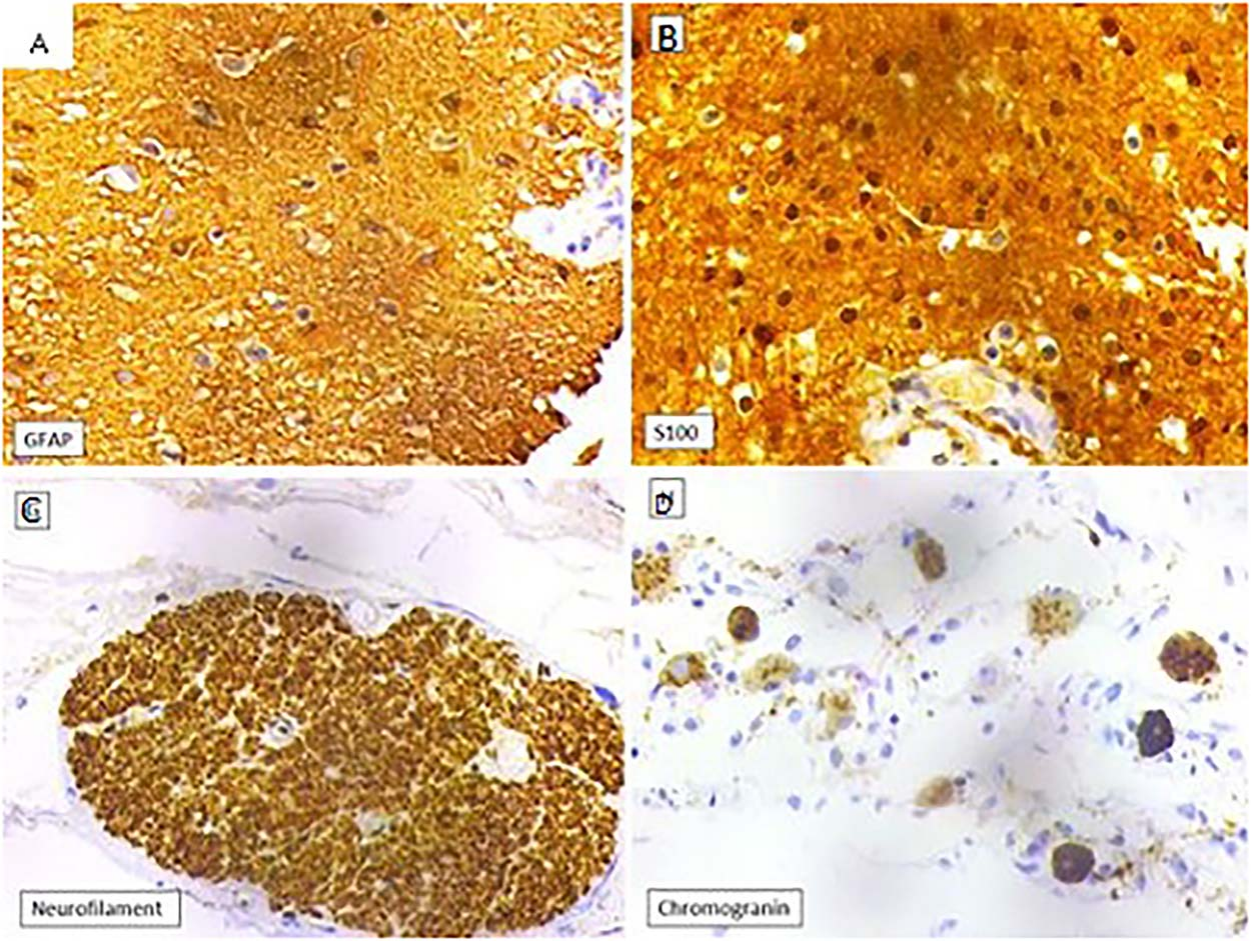
(A) Glial tissue showing GFAP positivity (IHC, 400×). (B) Glial tissue showing S100 positivity (IHC, 400×). (C) Nerve trunk showing neurofilament positivity (IHC, 100×). (D) Ganglion cells showing chromogranin positivity (IHC, 400×).

Histopathology showed cystic spaces lined by neuroglial tissue and a fibrocollagenous wall with focal areas showing nerve trunks and ganglion cells (Fig. 3A–D). No other embryonic tissue derivatives or teratomatous elements were seen. Immunohistochemistry showed strong GFAP and S100 positivity in the glial tissue. The nerve fibres showed positivity for neurofilament and the ganglion cells showed positivity for synaptophysin and chromogranin. Based on these findings, the diagnosis of NH was confirmed (Fig. 4A–D).

## Discussion

Neuroglial heterotopia is a rare, benign congenital anomaly characterized by presence of mature brain tissue in extracranial and extraspinal locations [1, 2]. Since the first report by Reid in 1852, there have been reports of this benign tumour occurring mostly in the head and neck regions [3]. Based on their location and histological characteristics, NHs can be classified as paraneuraxial or extraneuraxial, the latter type being more common [4–6]. Paraneuraxial tumours contain well-formed mature brain tissue and are located in paracranial or paravertebral regions. Extraneuraxial tumours, on the other hand, occur in superficial locations and contains disorganized neuroglial and mesenchymal tissues.

Although several theories have been put forward regarding aetiology of these tumours, none have been all encompassing to explain the different varieties reported so far. One theory proposes sequestration of herniated brain, which could explain the tumours with a connection with the spine [5, 6]. Other theories mention retention of vestigial neural crest cells [7], or aberrant migration and differentiation of neuroectodermal remnants [8]. Still others classified these tumours as hamartomas [9].

Neuroglial heterotopia in retroperitoneal location is very rare and only six cases have been reported in the English literature. Of these six cases, only one case was an infant [10]. Two cases were detected antenatally prior to 15 weeks of gestation and both were aborted [2, 5]. Three children did not have any symptoms except a lump in the abdomen at presentation. One six year old child presented with urinary tract infection [2]. These four cases were operated and none of them had any recurrence. Only two cases had connection with the spine [5, 6]. In the present case the tumour was of paraneuraxial type and had mostly cystic component with less solid tissue without any bone or cartilage.

Zhong *et al*. found high neuron specific enolase (NSE) level in their case of NH and suggested its role as a diagnostic and prognostic marker [2]. Histologically, NH is characterized by presence of neuroglial tissue consisting of mature astrocytes and glial fibres within a fibrocollagenous stroma, along with rare components like neurons, choroid plexus, and oligodendrocytes. Immunohistochemical staining for GFAP, S100, and NSE confirms the presence of glial tissue [6, 11]. The present case had neuroglial and fibrous tissue along with few nerve trunks and ganglion cells. Immunohistochemistry also confirmed the diagnosis. It is important to distinguish retroperitoneal NH from cystic neuroblastoma, which typically presents as a hypoechoic mass and exhibits prominent blood flow signals within the mass and may have small calcifications on contrast CT. Liver metastasis may also be seen [12].

Complete surgical excision of NHs is curative and 4%–10% cases can have recurrence [3, 13]. Although laparoscopic resection was an option in the present case, considering the large size and potential risk of spillage of cyst contents during laparoscopy, we decided to approach through a laparotomy.

## Conclusion

Retroperitoneal NH is an entity, rarely encountered during infancy. Pre-operative imaging, histopathology, and immunohistochemistry help in differentiating it from other common tumours at this age and location. A close follow-up is necessary after curative resection.
